# An unconditional prenatal income supplement is associated with improved birth and early childhood outcomes among First Nations children in Manitoba, Canada: a population-based cohort study

**DOI:** 10.1186/s12884-021-03782-w

**Published:** 2021-04-20

**Authors:** Jennifer E. Enns, Nathan C. Nickel, Mariette Chartier, Dan Chateau, Rhonda Campbell, Wanda Phillips-Beck, Joykrishna Sarkar, Elaine Burland, Alan Katz, Rob Santos, Marni Brownell

**Affiliations:** 1 Manitoba Centre for Health Policy, Department of Community Health Sciences, Rady Faculty of Health Sciences, University of Manitoba, 408-727 McDermot Ave, Winnipeg, Manitoba R3E 3P5 Canada; 2 First Nations Health and Social Secretariat of Manitoba, Winnipeg, Canada; 3 Department of Family Medicine, Rady Faculty of Health Sciences, University of Manitoba, Winnipeg, Canada

**Keywords:** Income supplement, Poverty, Birth outcomes, Early child development, Breastfeeding, Prenatal care, Early development instrument, Indigenous, First Nations

## Abstract

**Background:**

In Manitoba, Canada, low-income pregnant women are eligible for the Healthy Baby Prenatal Benefit, an unconditional income supplement of up to CAD $81/month, during their latter two trimesters. Our objective was to determine the impact of the Healthy Baby Prenatal Benefit on birth and early childhood outcomes among Manitoba First Nations women and their children.

**Methods:**

We used administrative data to identify low-income First Nations women who gave birth 2003–2011 (*n* = 8209), adjusting for differences between women who received (*n* = 6103) and did not receive the Healthy Baby Prenatal Benefit (*n* = 2106) with using propensity score weighting. Using multi-variable regressions, we compared rates of low birth weight, preterm, and small- and large-for-gestational-age births, 5-min Apgar scores, breastfeeding initiation, birth hospitalization length of stay, hospital readmissions, complete vaccination at age one and two, and developmental vulnerability in Kindergarten.

**Results:**

Women who received the benefit had lower risk of low birth weight (adjusted relative risk [aRR] 0.74; 95% CI 0.62–0.88) and preterm (aRR 0.77; 0.68–0.88) births, and were more likely to initiate breastfeeding (aRR 1.05; 1.01–1.09). Receipt of the Healthy Baby Prenatal Benefit was also associated with higher rates of child vaccination at age one (aRR 1.10; 1.06–1.14) and two (aRR 1.19; 1.13–1.25), and a lower risk that children would be vulnerable in the developmental domains of language and cognitive development (aRR 0.88; 0.79–0.98) and general knowledge/communication skills (aRR 0.87; 0.77–0.98) in Kindergarten.

**Conclusions:**

A modest unconditional income supplement of CAD $81/month during pregnancy was associated with improved birth outcomes, increased vaccination rates, and better developmental health outcomes for First Nations children from low-income families.

**Supplementary Information:**

The online version contains supplementary material available at 10.1186/s12884-021-03782-w.

## Background

The prenatal period is the first of several defining life phases that give shape to children’s health trajectory. Prenatal exposure to poverty and its correlates, which include higher maternal stress levels, higher likelihood of poor nutrition, and higher rates of smoking and substance use, can have long-lasting consequences for children [1–4]. Women living in poverty during the prenatal period are also more likely to experience adverse birth outcomes (including low birth weight and preterm birth) [5, 6], which contribute to many subsequent health, developmental and cognitive challenges as their children grow [7–10]. In recognition of these harmful sequelae, considerable public health efforts continue to attempt to counteract socioeconomic disadvantage among expectant mothers.

Programs providing income supplements (i.e., cash benefits or cash transfers) to low-income pregnant women are an increasingly common initiative, especially in low- and middle-income countries (LMICs) [11]. Eligibility for these income supplements is often conditional upon the expectant mother complying with pre-determined requirements, such as regular visits to a health facility for prenatal care. Well-studied conditional cash transfer programs in LMICs, including the Progresa [12] and Oportunidades [13] programs in Mexico, the Bolsa Familia program in Brazil [14, 15], and the Janani Suraksha Yojana program in India [16], are all associated with improved birth outcomes such as increased birthweight and decreased neonatal/infant mortality [17]. However, income supplement programs for low-income pregnant women living in *high-income* countries are relatively rare, despite widening health inequities in birth outcomes in many of these places [18].

In Canada, there are significant inequities in many types of health outcomes, including birth outcomes, between Indigenous people^1^ and other Canadians [19]. Understanding the disparities between Indigenous and non-Indigenous people requires an appreciation of the historical, political, societal and economic determinants that influence Indigenous health. For First Nations, these determinants include the multi-generational burden of trauma they carry from forced attendance at residential schools and the harms perpetrated by the ‘Sixties Scoop’, during which First Nations children were removed from their own families and placed in predominantly white families, leading to extensive loss of family connections, language and cultural identity [20, 21]. Colonial policies and practices have created many other social and economic barriers, which have denied many First Nations access to quality housing, education and employment opportunities, and have made it difficult for them to access healthcare, both for geographical reasons and due to inherent racism and a lack of cultural sensitivity in the healthcare system [22]. Collectively, these challenges make First Nations women and their children more likely than other Canadian women to experience adverse birth and early childhood outcomes; indeed, recent studies show that birth and early childhood outcomes in this population consistently fall well below Canadian norms [23, 24]. For example, in a population-based study of First Nations and Inuit birth outcomes in the Canadian province of Quebec, First Nations perinatal and infant mortality rates were 1.5 to 2 times higher than non-Indigenous rates [24]. In Manitoba, First Nations are 1.5 to 2 times more likely to have a preterm or large-for-gestational-age birth, and the likelihood of a First Nations newborn being readmitted to hospital is nearly twice as high as other Manitobans [25]. And in a nationally representative sample of First Nations, Metis and Inuit, First Nations rates of sudden infant death syndrome were reported to be more than seven times higher than in the rest of the Canadian population [23]. Evidence for interventions that can improve birth outcomes among First Nations populations is highly sought after, as evidenced by the Truth and Reconciliation Commission of Canada’s Calls to Action, which call upon the federal government to close the gaps in infant and child health outcomes between Indigenous and other Canadians [26].

In Manitoba, which is home to the largest proportion of First Nations people among the Canadian provinces, there is a unique opportunity to evaluate an intervention with the potential to improve birth and early childhood outcomes in this population. The Healthy Baby Prenatal Benefit (HBPB) is an unconditional income supplement available to all low-income pregnant women in Manitoba in their second and third trimesters. To be eligible for the benefit, applicants must provide proof of pregnancy and have an annual income of CAD $32,000 or less; once enrolled, they receive a monthly cheque of a maximum amount of CAD $81/month. Previous research on the whole-of-Manitoba population has shown that receipt of the HBPB is associated with improved birth outcomes (fewer low birth weight infants and preterm births, and higher breastfeeding initiation rates) when compared to birth outcomes among low-income mothers who would have been eligible for the benefit but did not apply [27, 28]. However, it is not yet known if the HBPB has the same beneficial effects for First Nations women and their families. The underlying mechanisms that are believed to improve outcomes for low-income families, such as increasing access to resources and health and social services [29], might not hold true for First Nations families, given the numerous challenges they face. In addition, Indigenous women and children are often under-represented in the health literature [30] – this study, which was undertaken in partnership with researchers from the First Nations Health and Social Secretariat of Manitoba, seeks to recognize and characterize inequities in Indigenous health and bring First Nations voices to the forefront. Our objective was to investigate whether the HBPB was associated with improved birth and early childhood outcomes specifically for Manitoba First Nations women and their children.

## Methods

### Study setting

The study was conducted in Manitoba, Canada (population ~ 1.4 M). Manitoba is broadly representative of other Canadian provinces on a number of key health and social indicators [31, 32]. However, Manitoba is unique in offering the HBPB, an unconditional prenatal income supplement for low-income women, and also in having the capacity to link individual-level information on receipt of the HBPB to extensive health and social administrative datasets. The study received approval from the University of Manitoba’s Health Research Ethics Board, the government of Manitoba’s Health Information Privacy Committee, and the Health Information Research Governance Committee of the Assembly of Manitoba Chiefs.

### Data sources

We used administrative data from the PATHS Data Resource [33], a collection of population-based, individual-level data describing health status, health service use and social service use for all children born 1984–2014 and registered for universal healthcare in Manitoba, and contained within the Population Research Data Repository at the Manitoba Centre for Health Policy. All records in the Data Repository are stripped of personal information (e.g., names and addresses), but are linkable at the individual level using a scrambled numeric identifier. The Repository data have been used extensively in research and their validity for population studies has been well documented [34, 35]. The specific datasets used in this study are described in Supplementary File 1.

### Study cohort

**Fig. 1:**
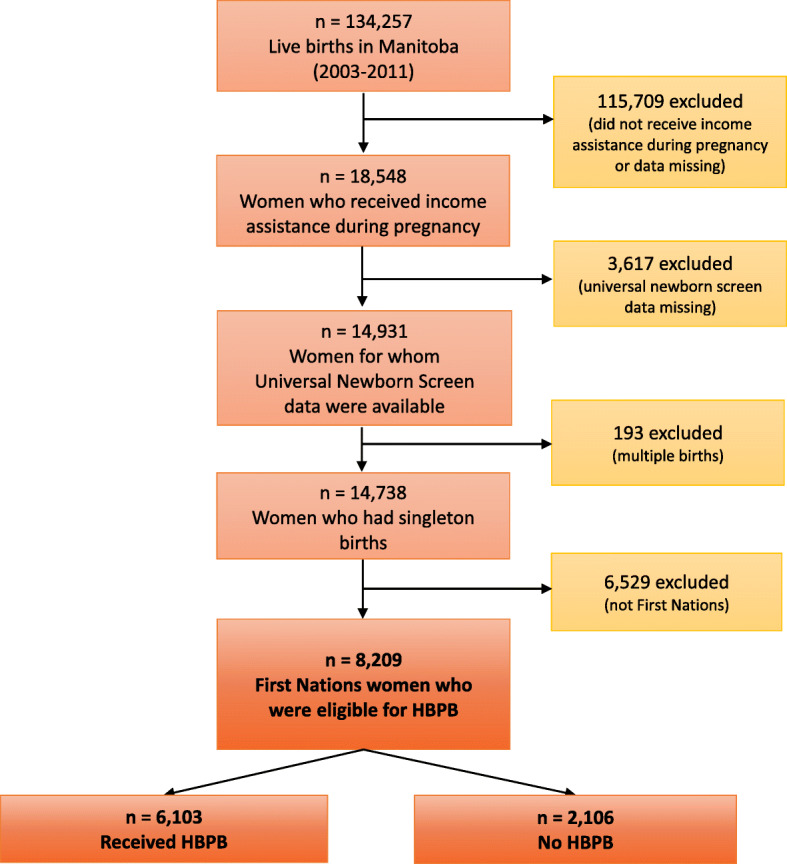
Study Cohort Development. We identified all Manitoba First Nations mothers with births from 2003 to 2011 who received income assistance during their pregnancies. HBPB: Healthy Baby Prenatal Benefit

The study cohort included all First Nations women in Manitoba who had a live birth between 2003 and 2011 and who were eligible to receive the HBPB. We compared First Nations women who applied for and received the benefit (*Received HBPB* group) to those who were eligible but did not receive the benefit (*No HBPB* group) (Fig. 1). Women who did not receive the benefit may not have known about the benefit or may have chosen not to apply for it, for example, because they believed they wouldn’t be eligible, because they encountered barriers in the application process, or because they were distrustful of the healthcare system or the child welfare system [29].

We formed the cohort by first identifying all women in Manitoba who had a live birth between 2003 and 2011, among whom we identified low-income women using information on receipt of income assistance during their pregnancy. Because income assistance records were used to identify low-income women, and because the province does not keep records of First Nations people living on-reserve who receive federal income assistance, most women living on-reserve were excluded from the study cohort. We also excluded women for whom we did not have data from Manitoba’s universal newborn screen; the information collected via the newborn screen (e.g., health-related behaviours like prenatal smoking or alcohol and drug use, and other information on maternal mental health, single parenthood and family functioning – the full list of variables is available in Table 1 and a copy of the screen is available in Supplementary File 2) was required to ensure we could satisfactorily balance characteristics between women who did and did not receive HBPB. Women who had multiple births were excluded due to the greater likelihood of poor health outcomes compared to singleton births. Finally, we selected First Nations women by cross-referencing five different datasets that contain information on First Nations identity: i) the Manitoba First Nations Research File; ii) data from the HBPB application form; iii) data from Manitoba’s Families First universal newborn screen; iv) income assistance data; and v) the Early Development Instrument data (full dataset descriptions in Supplementary File 1).

**Table 1:** Characteristics of Low-Income First Nations Women before and after Propensity Score Weighting

	Received HBPB *n* = 6103	No HBPB *n* = 2106	***P***	Standardized Diff Before Weighting	Standardized Diff After Weighting
Universal newborn screen completed prenatally^a^ (%)	10.73	6.41	< 0.001	4.32	0.00
Alcohol or substance use during pregnancy (%)	36.41	38.37	0.110	1.96	0.00
Smoked during pregnancy (%)	56.37	59.83	0.005	3.46	0.00
Did not complete high school (%)	59.07	58.59	0.703	0.48	0.01
Single parent family (%)	51.07	45.25	< 0.001	5.82	0.00
No prenatal care before 6 months (%)	8.67	17.90	< 0.001	9.23	0.00
Has mood or anxiety disorder (%)	23.63	19.37	< 0.001	4.26	0.00
Has schizophrenia (%)	0.88	0.66	0.303	0.22	0.01
Has a mental disability (%)	1.21	1.19	0.926	0.02	0.00
Family history of disability (%)	3.41	3.70	0.532	0.29	0.01
Father is antisocial (%)	5.21	4.23	0.060	0.98	0.01
Mother is antisocial (%)	2.11	2.61	0.206	0.50	0.01
Current substance use (%)	3.60	4.51	0.076	0.91	0.00
Social isolation (%)	6.77	7.74	0.144	0.97	0.00
Relationship distress (%)	16.24	14.62	0.074	1.62	0.01
Violence between parents (%)	9.27	8.31	0.172	0.96	0.01
Abused as a child (%)	20.27	17.33	0.003	2.94	0.01
Has diabetes (%)	3.24	1.76	< 0.001	1.48	0.00
Family lives on reserve (%)	2.64	4.61	< 0.001	1.97	0.00
Age at first birth					
Count	6103	2106			
Mean	18.7	18.5	0.013	0.20	0.01
Area-level SES index (higher value = lower SES)					
Count	6098	2104			
Mean	1.1	1.0	< 0.001	0.10	0.00

^a^Families First screens are typically completed postnatally, so when a screen is completed prenatally, this indicates the presence of additional risk factors. *HBPB* Healthy Baby Prenatal Benefit, *SES* socioeconomic status

### Variables

Our exposure variable was receipt of HBPB during pregnancy. The amount each family receives is calculated on a sliding scale according to annual family income before taxes. Preliminary analyses demonstrated that nearly all First Nations HBPB recipients in the study cohort received the maximum benefit and so we did not look for a dose-response effect. Information on birth outcomes (low birth weight, preterm birth, small- or large-for-gestational age, 5 min Apgar score, breastfeeding initiation, hospital readmission within 28 days of birth, and length of birth hospitalization) was obtained from hospital discharge abstracts (see Supplementary File 3 for details). Information on early childhood outcomes (childhood immunization at age one and two, and hospital admission in the first 2 years of life) was extracted from the Manitoba Immunization Monitoring System and hospital discharge abstracts, respectively. Developmental vulnerability was measured using the Early Development Instrument, a kindergarten teacher-completed questionnaire that assesses five developmental domains in kindergarten students [36]. Each domain is scored as ‘vulnerable’ or ‘not vulnerable’ at the 10th percentile according to national standards [37, 38].

We adjusted for potentially confounding variables with information from the universal newborn screen (Table 1). Using methods developed by Rubin (2001), we created propensity scores to adjust for potential systematic differences between the HBPB and No HBPB groups [39]. We used multiple logistic regression to create the propensity scores; receipt of HBPB was the dependent variable and the characteristics in Table 1 were covariates. Each woman’s propensity score indicates the probability that she received HBPB given her observed characteristics. From the propensity scores, we then developed inverse probability of treatment weights (IPTWs), which were used to reduce standardized differences between groups [40, 41].

### Statistical analyses

We ran generalized linear models with a binomial distribution for each of the dichotomous outcomes, and used the log-link function to estimate crude and IPTW-adjusted risk ratios associated with receiving HBPB. For continuous outcomes (birth hospitalization length of stay), we ran generalized linear models with a negative binomial distribution, and used the log-link function to estimate the mean length of stay associated with receiving HBPB by modeling the crude (unweighted) and IPTW-adjusted means. We measured sensitivity to unmeasured confounding using a gamma sensitivity test, which estimates the strength of any unmeasured confounder required to nullify statistically significant results [42].

Population-attributable fractions (PAFs) and population-preventable fractions (PPFs) were calculated to quantify the impact of the HBPB [43]. Where HBPB was associated with an increase in the outcome, we used the following formula: PAF = Pe × [(RR – 1)/RR], where Pe is prevalence of the exposure. Where HBPB was associated with a reduction in the outcomes, we used: PPF = Pe × (1-RR). Confidence intervals were calculated using the standard deviation of a bootstrapped mean PAF or PPF derived from 500 samples of the population.

## Results

The final study cohort included 8209 First Nations women who gave birth to live singleton infants between 2003 and 2011, who received income assistance during their pregnancy, and for whom we had data from the universal newborn screen. Among these, 6103 women received the HBPB, while 2106 women did not receive the benefit (Fig. 1). The women’s individual and family characteristics are shown in Table 1. We initially observed some significant differences between the two groups on several characteristics. For example, women in the *Received HBPB* group were more likely to be single parents than women in the *No HBPB* group, whereas women in the *No HBPB* group were more likely to have reported that they smoked during their pregnancy than women in the *Received HBPB* group. After applying the IPTWs, all the covariates’ standardized differences dropped to 0.01% or less, indicating that the groups’ characteristics were satisfactorily balanced [44].

In Table 2, we present the crude rates, unweighted means and adjusted risk ratios of birth outcomes among First Nations infants whose mothers did or did not receive HBPB. Receiving HBPB was associated with a significant reduction in low birth weight and preterm births, and a significant increase in breastfeeding initiation. There was no association between HBPB and small-for-gestational-age or large-for-gestational-age births, low 5-min Apgar scores, hospital readmission within 28 days of the birth, or length of hospital stay following birth. For low birth weight births and preterm births, the γ sensitivity values of > 40 indicate that the findings are robust to unmeasured confounding. This means that in addition to the confounders we adjusted for in the propensity score, any unmeasured confounder would need to both correctly predict receipt of HBPB and account for ~ 40–50% of the relationship between receipt of HBPB and these two outcomes; the likelihood of such a confounder existing is very small. The robustness of the findings for breastfeeding initiation (with a γ sensitivity value of 20.1) could be somewhat more sensitive to unmeasured confounding.

**Table 2:** Association between Healthy Baby Prenatal Benefit (HBPB) and Birth Outcomes for First Nations Mothers and Infants

**Outcome**	***n***		**Crude Rates (%)**		**Adjusted Risk Ratio**	**95% CI**	***P***	**γ Sensitivity Value**^a^
	**HBPB**	**No HBPB**	**HBPB**	**No HBPB**				
Low Birth Weight	302	151	4.95	7.17	0.741	0.621, 0.883	< 0.001	41.2
Preterm Birth	537	249	8.80	11.82	0.774	0.679, 0.883	< 0.001	48.5
Small for Gestational Age	402	162	6.59	7.70	0.916	0.781, 1.073	0.276	–
Large for Gestational Age	1216	367	19.93	17.45	1.069	0.976, 1.170	0.151	–
Low 5-min Apgar Score	183	69	3.00	3.28	0.870	0.681, 1.111	0.265	–
Breastfeeding Initiation	3765	1191	61.72	56.55	1.046	1.009, 1.085	0.014	20.1
Hospital Readmission within 28 Days	159	43	2.76	2.22	1.163	0.871, 1.554	0.306	–
**Outcome**	***n***		**Unweighted Means**		**Weighted Means (95% CI)**		***P***	**γ Sensitivity Value**
	**HBPB**	**No HBPB**	**HBPB**	**No HBPB**	**HBPB**	**No HBPB**		
Birth Hospitalization LOS in days (for vaginal births only)	5260	1796	2.96	3.05	2.96 (2.87, 3.04)	3.04 (2.93, 3.15)	0.239	–

^a^The γ sensitivity analyses were conducted only for findings that were statistically significant. *HBPB* Healthy Baby Prenatal Benefit, *LOS* length of stay

Table 3 presents the crude rates and adjusted risk ratios of early childhood outcomes among First Nations children whose mothers did or did not receive HBPB. Receiving HBPB was associated with a significantly higher likelihood of children having a complete set of immunizations at age 1 and 2, and a significantly lower likelihood that children would be developmentally vulnerable in the domains of ‘language and cognitive development’ and ‘general knowledge and communication skills’ in kindergarten. There was no association between HBPB and hospital admission in the child’s first 2 years of life or for any of the other three developmental domains on the Early Development Instrument. For the immunization outcomes, the γ sensitivity values of > 60 indicate that the findings are robust to unmeasured confounding. In addition to adjusting for the confounders in the propensity score, any unmeasured confounder would need to both correctly predict receipt of HBPB and account for ~ 60–70% of the relationship between receipt of HBPB and these outcomes; and again, the likelihood of such a confounder existing is very small. The robustness of the findings for developmental vulnerability at Kindergarten (with γ sensitivity values of 11.0–13.6) is somewhat more likely to be sensitive to unmeasured confounding.

**Table 3:** Association between Healthy Baby Prenatal Benefit (HBPB) and Early Childhood Outcomes among First Nations Children

Outcome	***n***		Crude Rates (%)		Adjusted Risk Ratio	95% CI	***P***	γ Sensitivity Value
	HBPB	No HBPB	HBPB	No HBPB				
Complete Childhood Vaccinations (Age 1)	4063	1240	67.48	60.14	1.097	1.059, 1.137	< 0.001	61.6
Complete Childhood Vaccinations (Age 2)	2964	852	49.79	41.74	1.189	1.130, 1.250	< 0.001	70.9
Hospital Readmission before Age 2	1166	410	42.10	41.45	0.979	0.893, 1.073	0.648	–
Developmental Vulnerability at Kindergarten								
Physical Health and Well-Being	658	230	30.45	33.00	0.927	0.831, 1.034	0.173	–
Social Competence	579	172	26.78	24.68	1.075	0.948, 1.219	0.262	–
Emotional Maturity	467	143	21.61	20.58	1.030	0.892, 1.188	0.690	–
Language and Cognitive Development	619	226	28.64	32.42	0.879	0.786, 0.983	0.023	13.6
Communication Skills and General Knowledge	531	191	24.56	27.40	0.870	0.769, 0.984	0.027	11.3
One or more domains	1180	387	54.58	55.52	0.982	0.919, 1.050	0.595	–

*EDI* Early Development Instrument, *HBPB* Healthy Baby Prenatal Benefit

**Fig. 2:**
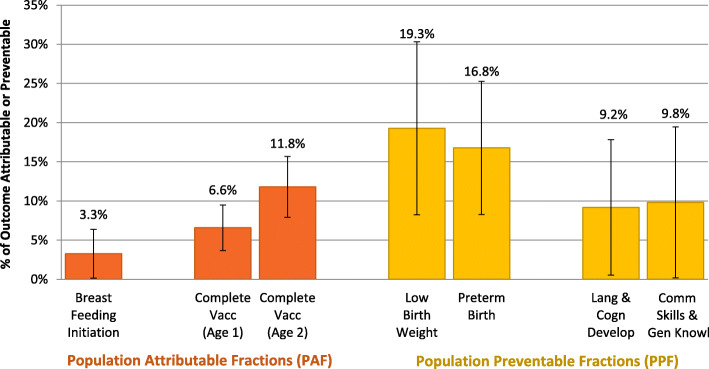
Population Attributable and Population Preventable Fractions of Outcomes Associated with the Healthy Baby Prenatal Benefit. Vacc: vaccination; Lang & Cogn Develop: language and cognitive development score on the Early Development Instrument; Comm Skills & Gen Knowl: communication skills and general knowledge score on the Early Development Instrument

Figure 2 illustrates the PAF for breastfeeding initiation (3%, although the error bars indicate that this was not statistically significant), and complete childhood vaccination at age 1 and 2 (6.6 and 11.8%, respectively). The PPF for low birth weight was 19.3%, for preterm birth was 16.8%, and for developmental vulnerability at kindergarten was 9.2% (for language and cognitive development) and 9.8% (for general knowledge and communication skills).

## Discussion

This retrospective cohort study on a prenatal income supplement provided to low-income First Nations women demonstrates improvements in several birth and early childhood outcomes. Receiving the HBPB was associated with reductions in low birth weight births and preterm births, translating into 19% of low birth weight births and almost 17% of preterm births prevented among First Nations women. As well, receiving the HBPB was associated with a higher likelihood of First Nations children receiving a complete series of vaccinations at age 1 and 2, of which 6.6 and 11.8% (respectively) can be attributed to the benefit.

The finding that a prenatal income supplement of only CAD $81/month is associated with improved birth outcomes is remarkable, given that it is well known that influencing clinical outcomes within lower socioeconomic conditions has proven very difficult. Many public discourses and policy interventions focus attention on the individual behaviours of pregnant women (such as diet and exercise) [45], but in a real-world context that recognizes the close relationship between low socioeconomic status and disparities in health, the fundamental causes of adverse health outcomes must be addressed if there is to be any hope of improving birth outcomes at the population level. This perspective is emphasized by Hughes and Simpson (1995), who argue that reducing persistent disparities in low birth weight requires embracing a broader definition of health that incorporates multiple social dimensions [46]. Further evidence on the mechanisms by which an unconditional prenatal income supplement can bring about improved clinical outcomes is presented by Struthers et al. [29], who interviewed recipients of the HBPB about what they used the benefit for and how it impacted them. Participants in that study described how the money helped them purchase items to prepare for the birth of their child, improve their nutrition, and engage in self-care behaviours that reduced their stress. Given these findings and the results of other cash transfer programs [12–16], and taking into account the large health inequities in birth outcomes between First Nations and other Canadians [47–49], there is a clear need to ensure adequate investment in resources for low income First Nations women and their families. Notably, in the nearly 20 years the HBPB has been available, the amount of the benefit has not increased at all. Whether more money could result in even better outcomes remains to be investigated.

Another important finding of the study was that First Nations children whose families received the HBPB were more likely than children from families who did not receive the benefit to have all of the recommended vaccinations at age one and age two. Vaccination is a vital component of preventive healthcare in young children that depends on high levels of coverage. While vaccination rates among First Nations children, particularly those living on-reserve, used to be lower than the general Canadian population [50], recent data suggest that the percent of First Nations infants and children who are fully vaccinated has increased in recent years and is not substantially different from average provincial and national estimates in Canada [51]. However, the percentage of children who are fully vaccinated by age 2 still remains well below the target of 95% in many jurisdictions, including Manitoba. Many First Nations mothers recognize the importance of vaccines in preventing childhood disease, but some families may be hesitant to have their children vaccinated because of a history of negative interactions with the healthcare system, or because of difficulty accessing a clinic [50]. Families living in rural or remote communities may not have a health centre nearby, and some clinics may have staffing shortages and high rates of turnover, further disrupting health service delivery and monitoring of public health measures like vaccination [52]. In the context of our study, the HBPB may seem like an unlikely public health strategy for increasing vaccination rates; however, we speculate that the benefit, and the larger Healthy Baby program of which it is a component, could play a role in connecting families to primary care providers during and after pregnancy, and this may contribute to ensuring that First Nations children are protected from vaccine-preventable illnesses.

Our study’s findings on the Early Development Instrument scores showed that First Nations children whose families received the HBPB were less likely to be developmentally vulnerable (in two developmental domains) when they entered kindergarten. It is notable that the two domains recipients of the HBPB were stronger in, ‘language & cognitive development’ and ‘general knowledge & communication skills’, are closely tied to academic achievement. Whether it is plausible that CAD $81/month during the latter two trimesters of a low-income woman’s pregnancy can have an impact on their child’s readiness for school is open to speculation – however, a clear gradient between household income and a child’s developmental vulnerability has been observed [53], and the HBPB represented a significant increase of about 10% in recipients’ average income. Another explanation for these findings is that, as the relatively low gamma sensitivity values for these outcomes indicate, there are other unmeasured factors at play that we were not able to account for in our analysis. The association between receiving the HBPB and being less likely to be developmentally vulnerable may only reflect the fact that some low-income households eligible for the benefit are already well-connected to health and social services and are providing high quality parenting to their young children during the early years – this is however a difficult concept to measure using administrative data.

### Strengths and limitations

Elements of our study design, including use of the comprehensive population-based Manitoba Population Research Data Repository to identify eligible First Nations women who were and were not receiving the HBPB, and use of IPTW and γ sensitivity analyses to balance the study groups and strengthen our confidence in the comparisons drawn between them, are strengths of this study. Using administrative data also allowed us to avoid problems associated with reporting and recall biases [54].

Despite these strengths, we were limited by potentially confounding factors that the data and our efforts to minimize bias could not account for. Although our use of propensity scoring minimized differences between the two study groups, there is still potential for selection bias in our study design due to intrinsic differences in the women who apply for the HBPB and those who do not. Some of the reasons why Manitoba women may not apply for the benefit were explored previously [29]. Another important limitation of the study was the need to limit the study cohort to women receiving provincial income assistance because we did not have access to the whole-population information on family income needed to calculate national measures like the Low Income Measure (LIM) or the Low Income Cut-Off (LICO). Our approach ensured that women in both study groups had equally low incomes, but we recognize that focusing on this very low income group may limit the generalizability of our findings. Our decision to limit the study cohort to women receiving income assistance also meant that we excluded most First Nations women living on-reserve, because income assistance data in Manitoba are only available for First Nations residents living off-reserve. Although we found that First Nations women living on reserve made up less than 40% of all First Nations women who gave birth during the study period, and that only 56% of these women applied for the HBPB, there are significant differences in health outcomes and income levels between women living on and off reserve that could influence the results of this study. A recent study on the health status of Manitoba First Nations reports that individuals living on reserve have higher premature mortality rates, lower rates of primary care visits but higher rates of hospitalization, and higher rates of mood and anxiety disorders compared to individuals living off reserve [55]. Data from the Canada Census and self-reported household income data place the majority of First Nations people living on-reserve in the lowest income quintiles and categories, compared to other Manitobans [55, 56]. Given the health and income disparities between those living on and off reserve, were we to include women living on reserve in future analyses, the HBPB would likely be associated with even more robust improvements in outcomes in this population.

## Conclusion

This study showed that the Healthy Baby Prenatal Benefit, an unconditional monthly benefit provided to low-income First Nations women during pregnancy, was associated with improved birth outcomes, higher rates of child vaccination, and a higher likelihood that kindergarten-aged children would be better prepared for school entry. That this population-level intervention was associated with better outcomes from infanthood through early childhood is remarkable, demonstrating how even modest financial support provided directly to First Nations families experiencing poverty without any “strings attached” can contribute to an upwards trajectory. The study findings align with what advocates have been saying for many years – that at least part of the solution to poverty is simply to provide sufficient resources to families experiencing poverty and allow them to determine how they should be used.

## Supplementary Information

**Additional file 1: Supplementary File 1.** List of Datasets from the Manitoba Population Research Data Repository. **Supplementary File 2.** Families First Screening Form. **Supplementary File 3.** List of Study Outcomes

## Data Availability

Data used in this study were derived from administrative health and social data as a secondary use. The data were provided to the Manitoba Centre for Health Policy (MCHP) under specific data sharing agreements only for approved use at MCHP. The original source data is not owned by the researchers or MCHP and as such cannot be provided to a public repository. However, the original data source and approval for use have been noted in the acknowledgments of the article, and where necessary, source data specific to this article or project may be reviewed at MCHP with the consent of the original data providers, along with the required privacy and ethical review bodies’ approval.

## Abbreviations

HBPB: Healthy Baby Prenatal Benefit
IPTW: Inverse Probability Treatment Weight
LICO: Low Income Cut-Off
LIM: Low Income Measure
LMIC: Low and Middle Income Countries
PAF: Population Attributable Fraction
PPF: Population Preventable Fraction

## Footnotes

1 Indigenous Peoples in Canada include First Nations, Inuit and Métis.
