# Supplementary material for: An unconditional prenatal income supplement is associated with improved birth and early childhood outcomes among First Nations children in Manitoba, Canada: a population-based cohort study
Source: BMC Pregnancy Childbirth. 2021 Apr 20;21:312. doi: 10.1186/s12884-021-03782-w (PMC8059008; doi:10.1186/s12884-021-03782-w)
Supplement: Supplementary file 1 — Additional file 1: Supplementary File 1. List of Datasets from the Manitoba Population Research Data Repository. Supplementary File 2. Families First Screening Form. Supplementary File 3. List of Study Outcomes [file 12884_2021_3782_MOESM1_ESM.docx]

**Supplementary Files**

**Manuscript Title:** An Unconditional Prenatal Income Supplement is Associated with Improved Birth and Early Childhood Outcomes among First Nations Children in Manitoba, Canada: A Population-Based Cohort Study

**Authors:** Jennifer E. Enns, Nathan Nickel, Mariette Chartier, Dan Chateau, Rhonda Campbell, Wanda Phillips-Beck, Joykrishna Sarkar, Elaine Burland, Alan Katz, Rob Santos, Marni Brownell

**Supplementary File 1**.

List of Datasets from the Manitoba Population Research Data Repository p. 2

**Supplementary File 2**.

Families First Screening Form **p. 3**

**Supplementary File 3**.

List of Study Outcomes p. 5

| **Supplementary File 1. List of Datasets from the Manitoba Population Research Data Repository** | |
| --- | --- |
| **Dataset** | **Description** |
| ***Datasets used to identify and describe individuals in the study cohort*** | |
| Manitoba Health Insurance Registry | Demographic information on Manitoba residents registered for universal healthcare |
| Canada Census | Small geographical area-level data from the Canada Census, used to create the Socioeconomic Factor Index 2 (SEFI 2), an index of socioeconomic status* |
| Manitoba First Nations Research File | An official record of persons registered as First Nations individuals under the Indian Act. |
| Families First Universal Newborn Screen Data | Information on biological, social and demographic factors of Manitoba families, including parents’ ethnic identity, history of alcohol and drug use, mental health disorders, and education. |
| Healthy Baby Prenatal Benefit Data | Information on biological, social and demographic factors of Manitoba families, including income, marital status, and benefits received. |
| Early Development Instrument | The Early Development Instrument assesses children’s developmental vulnerability across five domains through a kindergarten teacher-administered questionnaire. |
| Employment and Income Assistance Data | Demographic information on Manitoba residents who receive financial assistance. |
| Hospital Discharge Abstracts | Demographic and clinical information on hospitalized patients, including information on births and birth outcomes. |
| ***Datasets used to examine birth and early childhood outcomes*** | |
| Hospital Discharge Abstracts | Demographic and clinical information on hospitalized patients, including information on births and birth outcomes. |
| Manitoba Immunization Monitoring System | An electronic registry that captures pediatric and adult immunizations. |
| Early Development Instrument | The Early Development Instrument assesses children’s developmental vulnerability across five domains through a kindergarten teacher-administered questionnaire. |
| *Chateau D, Metge C, Prior H, Soodeen RA. 2012. Learning from the census: The Socioeconomic Factor Index (SEFI) and health outcomes in Manitoba. Can J Public Health 103(8 Suppl 2): S23-7. | |

**Supplementary File 2. Families First Screening Form**

| **Supplementary** **File 3. List of Study Outcomes** | |
| --- | --- |
| **Outcome** | **Definition** |
| Low birth weight | <2500 g at birth |
| Preterm birth | <37 week gestational age |
| Small for gestational age | <10^th^ percentile for gestational age and gender using Canadian standard^a^ |
| Large for gestational age | >90^th^ percentile for gestational age and gender using Canadian standard^a^ |
| 5-min Apgar score | Apgar score dichotomized into ≤7 and ≥8 |
| Breastfeeding initiation | Exclusive or partial breastfeeding at hospital discharge |
| Hospital readmission | Readmission to any Manitoba hospital within 28 days or 2 years of birth |
| Length of stay of birth hospitalization | Length of hospital stay at birth (continuous measure calculated separately for infants delivered vaginally and by caesarean delivery due to different stays expected for these groups) |
| Complete childhood immunization | Received complete series of recommended vaccinations from birth to age 1 and 2 using the Manitoba immunization schedule^b^ |
| Developmental vulnerability at kindergarten | Readiness in five developmental domains in Kindergarten, as measured by the Early Development Instrument: 1) physical health and well-being, 2) social competence, 3) emotional maturity, 4) language and cognitive development, and 5) communication skills and general knowledge |
| ^a^ Kramer et al. (2001).  ^b^ <https://www.gov.mb.ca/health/publichealth/cdc/div/schedules.html#child> | |
